# Comfort Need of Hospitalised Patients With Covid‐19 During Isolation Precaution: A Qualitative Study

**DOI:** 10.1002/nop2.70075

**Published:** 2024-11-01

**Authors:** Chiyar Edison, Agung Waluyo, Sri Yona, Tris Eryando

**Affiliations:** ^1^ Department of Medical Surgical Nursing, Faculty of Nursing Universitas Indonesia Kota Depok Jawa Barat Indonesia; ^2^ Nursing Department Universitas Indonesia Hospital Kota Depok Jawa Barat Indonesia; ^3^ Faculty of Public Health Universitas Indonesia Kota Depok Jawa Barat Indonesia

**Keywords:** Covid‐19, nurses, patients, professionalism

## Abstract

**Aim:**

This study aimed to explore the experiences of hospitalised patients with Covid‐19 in terms of the meeting of their comfort needs and the source of discomfort during isolation precautions.

**Design:**

Qualitative descriptive phenomenological approach was applied in this study.

**Methods:**

There were 16 hospitalised patients with Covid‐19 and 11 nurses who were thoroughly interviewed. Content analysis was conducted to examine the data of the interviews and then managed using Atlas ti‐9 software.

**Results:**

Five central themes were identified that describe the comfort needs and the source of discomfort, as follows: (1) nursing professionalism; (2) physical discomfort; (3) psychological responses; (4) poor sleep quality; and (5) spiritual adaptations.

**Public Contribution:**

The study promoted further explanation about comfort need in patients with Covid‐19 in isolation room. This study also highlighted that nursing care played a pivotal part in assisting patients to reach their highest comfort level.

## 1 Introduction

Since the end of 2019, there have been Covid‐19 outbreaks throughout the world. Indonesia is the largest country in Southeast Asia, which also has been affected since the first case was confirmed on 2 March 2020. During this study's data collection period (January–March 2022), the Health Ministry of Indonesia reported there had already been 40,858 deaths caused by Covid‐19 (Kemenkes 2021). In this period, Indonesia experienced a third wave of disease that was dominated by the Omicron variant. This situation led all hospitals in the country to increase the number of isolation wards required for quarantining patients hospitalised with Covid‐19 (CDC 2020). Therefore, nursing care for Covid‐19 patients in isolation wards has been in increasingly high demand.

## 2 Background

Isolation precautions have been identified to have several issues regarding negative impacts on patients (Guilley‐Lerondeau et al. 2017; Purssell, Gould, and Chudleigh 2020). The literature has shown that patients in isolation rooms experience psychological disorders such as anxiety as well as poor relationships with health workers (Guilley‐Lerondeau et al. 2017). Additionally, changes in patient behaviour and patient safety have been identified as related concerns (Sharma et al. 2020).

Previous studies have reported on the occurrence of anxiety, depression and sleep disorders in hospitalised patients with Covid‐19 during isolation precaution periods (Mazza et al. 2020; Zandifar et al. 2020). These psychological impacts occur along with reported physical issues such as dyspnoea, fever, sore throat and cough (CDC 2020). The impacts of the treatment of Covid‐19 patients in isolation rooms are not solely based on the progression of the disease—the effects also arise from the patients' need for special attention to address the issues related to the isolation itself. Therefore, the need for a holistic nursing care approach is urgently needed for Covid‐19 patients during isolation precaution periods.

The idea of comfort is a popular concept circulating in current holistic nursing literature. Kolcaba has explained that holistic comfort has the following four dimensions: physical, psycho‐spiritual, environmental and socio‐cultural (Kolcaba 1994). Holistic comfort is a useful indicator in measuring how nursing therapy can improve patient's quality of life (Yücel, Arslan, and Bagci 2020). Additionally, the literature showed that comfort is an essential need for patient during their hospitalisation (Nural and Alkan 2018), and an important outcome of nursing interventions (Yücel, Arslan, and Bagci 2020). In Indonesian population, several studies also revealed that comfort was an important aspect in nursing care (Jadmiko et al. 2022; Noor, Maria, and Agianto 2016). It has been shown that comfort need should be also considered as the outcome of nursing intervention in patient with Covid‐19 during isolation precaution.

The discomfort experiences of patients with Covid‐19 in Indonesia were probably caused by several clinical manifestations of disease and environment of isolation room (Aungsuroch, Juanamasta, and Gunawan 2020). Moreover, comfort has been an emerging word in several quotes of previous qualitative studies findings that explored the experience of hospitalised patient with Covid‐19 in Taiwan and China. The ‘comfort’ and ‘discomfort’ words were mentioned by some informants when they shared their experiences of discomfort related with Covid‐19 symptoms and healthcare service during isolation care (Hsiao et al. 2021). However, there was no further explication related to the characteristics of comfort needed in both studies. Additionally, based on the author's experience in providing nursing care to patient with Covid‐19 and frontier health personnel within patients, they expressed their discomfort experience due to their physical and mental condition during isolation precaution.

Accordingly, there should be an investigation to explore the experience of patients with Covid‐19 related with their comfort need and the cause of discomfort. In order to improve quality of nursing care holistically, it is imperative to explore how hospitalised patients with Covid‐19 perceive their comfort needs as being met. The present study aimed to explore the experiences of hospitalised patients with Covid‐19 in terms of how their comfort needs were met and the source of discomfort during isolation precautions.

## 3 Methods

### 3.1 Study Design

This study was conducted utilising a qualitative descriptive phenomenological design. This design was chosen because the study looks at the complex phenomenon of patients with Covid‐19 during isolation precautions, particularly in terms of their experience of comfort. A qualitative descriptive phenomenological approach has a design that is useful in exploring participants' experiences or perspectives on a given circumstance (Loiselle and McGrath 2011).

### 3.2 Population and Sample

This study involved participants who encompassed hospitalised patients with Covid‐19 and nurses treating these patients. Though this study explored the experience of patients, nurses were involved as informants to confirm information obtained from patient participants. All participants were recruited from (Universitas Indonesia Hospital). This hospital was chosen due to its status as the main referral hospital for the case of Covid‐19 in Depok city. This study used a purposive sampling method to ensure the participant criteria were appropriate for the objective of the study.

### 3.3 Inclusion and Exclusion Criteria

The eligible participants were invited by the researcher and head nurses based on meeting the inclusion criteria. The inclusion criteria of patients were as follows: patient with confirmed moderate Covid‐19, older than 18 years of age and placement in an isolation precaution area in the hospital. For participant nurses, the inclusion criteria included nurses who treated patients with Covid‐19 and minimum bachelor's degree. The exclusion criterion of participant patients was a patient with a deterioration of clinical signs. In total, there were 27 participants, comprised of 16 patients and 11 nurses.

### 3.4 Data Collection

Data were collected by conducting semi‐structured and in‐depth interviews from December 2021 to March 2022. Interviews were undertaken using mobile phones due to quarantine guidelines of social distancing. When the eligible participants, both patients and nurses, had been determined, the researcher arranged interview times with all parties. Afterwards, interviews were undertaken through video‐telephone calls. Ensuring the bracketing applied during all interview process, all participants must not have prior relationship with interviewer.

All the interviews were performed by the first author, who is a doctoral candidate student. Both the second and the third authors are PhD‐level scientists in the medical surgical nursing area. The fourth author is a scientist in the public health area. The first, second and fourth author are male (CE, AW, TE), and the third author are female (SY). These researchers have no prior experience in taking care of patients with Covid‐19 in the isolation room. Two researchers (AW, SY) are highly experienced in conducting qualitative research. The preparation of researchers prior to the interview process included constructing a question list and conducting pilot interview.

The interview process was guided by a predetermined question that was established through a literature review and consultation with a professor and an associate professor with expertise in qualitative methodology. For patient interviews, the question list included the following prompts: please share your experience during isolation precautions with Covid‐19; how do you define comfort based on your experience during isolation precautions?; please provide any examples of experiences of comfort or discomfort; and what do you expect from nurses in terms of fulfilling your comfort needs? In terms of the nurse interviews, the question list was as follows: please share your experience in caring for patients with Covid‐19 in isolation; what do you think the comfort needs of patients with Covid‐19 during isolation precautions include?; and how did you fulfil the comfort needs of patients with Covid‐19 during isolation precautions?

The interviews were recorded via smartphone. Each interview lasted from 30 to 40 min. Afterwards, they were transcribed verbatim and coded by the researcher to analyse and extract information. To identify the focus of research and data saturation, transcribing process was carried out on the day of interviews.

There was not a single informant who rejected to participate in this study or dropped out during the interview process. Saturation was assumed to have occurred after 16 patients and 11 nurses were interviewed. The saturation point was determined based on the research questions. Therefore, data collection was ceased when no new themes emerged from the accumulated interview data.

### 3.5 Data Analysis

Content analysis was conducted to examine the interview data. This approach is deemed suitable to analyse the multifaceted and important phenomena of nursing (Vaismoradi, Turunen, and Bondas 2013). Graneheim and Lundman's method was applied in the data analysis which was conducted based on three phases: preparation, organising and reporting phase (Graneheim and Lundman 2004; Vaismoradi, Turunen, and Bondas 2013).

Preparation phase included obtaining the sense of whole data and determining the unit of analysis. In this phase, the interview transcripts were reviewed multiple times by all researchers to identify and clarify meaningful statements about their experiences with meeting comfort needs, whether as a patient or nurse.

Subsequently, the organising phase was managed by determining codes, creating categories and deciding final themes. All the researchers independently identified repeated concepts with similar meaning in the verbatim transcriptions to put them under the same codes. These codes were categorised to the same category label. Afterwards, all researchers put all categories into discussion to reach consensus on final themes. All data management process was assisted by using Atlas ti‐9 software. Lastly, in the reporting phase, selective quotes were determined to represent each theme.

### 3.6 Ethical Considerations

This study was reviewed and approved by the Institutional Review Board (IRB) of university teaching hospital where the authors were affiliated with the following issued number: 0062/SKPE/KKO/2021/00. Before the interviews, all participants provided both verbal and written information regarding participation in the study and agreed to participate by signing an informed consent form. Participants were treated with respect during the interview process. The research team assured participants that no identifying information regarding the informants would be used. Participants were informed that they were permitted to abort the interview process without providing a reason for doing so. Moreover, all data were appropriately stored on a hard disk drive and password protected.

### 3.7 Rigour

The four criteria established by Lincoln and Guba were applied to ensure the rigour of this study, namely credibility, transferability, dependability and conformability (Lincoln and Guba 1985). Firstly, credibility was achieved through conducting pilot interviews to test the protocol interview and returning research findings to three participants to confirm the reliability and accuracy of data analysis. Secondly, transferability was ensured by valuing the diversity of age, gender and marital status of patients. Thirdly, to establish dependability, study design and data analysis were explicated in detail. Finally, conformability was facilitated by conducting a reflective analysis among research members before the interview and involving all authors during data analysis simultaneously. To strengthen the results of data analysis of patient interviews, nurses were involved in the interview process due to their role in meeting the patient's need.

## 4 Findings

In total, 27 individuals participated in the study, including 16 participant patients (PPs) and 11 participant nurses (PNs) who provided care for Covid‐19 patients. The 16 patients included seven male participants (43.8%) and nine female participants (56.3%). The age of the patients ranged from 24 to 52 years old. The occupations of the patients included the following: 12 private employees (75%), two civil servants (12.5%), one entrepreneur (6.3%) and one person who identified as a housewife (6.3%). In terms of educational background, most of the patients were undergraduate graduates, with 13 people (81%) holding such degrees; the other three (19%) held diplomas. In terms of marital status, there were 10 married patients (62.5%) and six unmarried patients (37.5%). Most of the patients were Muslims (94%), with only one person belonging to the Christian faith (6%) (Table 1).

**TABLE 1:** Characteristics of patient participants (*N* = 16).

Variables	Frequency	Percent
Gender		
Men	7	43.8
Women	9	56.3
Age (years)		
Youth (18–44)	15	93.8
Middle age (45–63)	1	6.3
Occupation		
Private‐employee	12	75
Civil‐servants	2	12.5
Entrepreneur	1	6.3
Housewife	1	6.3
Educational background		
Undergraduate	13	81
Diploma	3	19
Married status		
Married	10	62.5
Single	6	37.5
Religion		
Moslem	15	93.8
Christian	1	6.3

The nurse participants were all female (100%). The average age of the nurses was 26 years. All the nurses had graduated with a bachelor's degree. Six nurses had taken care of patients with Covid‐19 for more than 1 year. All nurses were Muslim and unmarried. Throughout their experience treating Covid‐19 patients, five nurses indicated that they had taken care of Covid‐19 patients for more than 1 year (45.4%); and the rest of the nurses had treated such patients for less than 1 year (54.6%) (Table 2).

**TABLE 2:** Characteristics of nurse participants (*N* = 11).

Variables	Frequency	Percent
Gender		
Men	0	0
Women	11	100
Age (years)		
Youth (18–44)	11	100
Middle age (45–63)	0	0
Educational background		
Bachelor	11	100
Diploma	0	0
Duration of Covid‐19 experience		
< 1 year	5	45.4
> 1 year	6	54.6

### 4.1 Five Themes

Table 3 shows the themes and categories that were extracted from data analysis. Each theme is explained below along with its categories and quotations of informants.

**TABLE 3:** Themes and categories.

Categories	Themes
The fulfilment of basic need	The nursing professionalism
The attitude of nurses	
Clinical manifestation disease	Physical discomfort
The impact of the therapy programme	
Anxiety	Psychological responds
Self‐acceptance of disease	
Clinical manifestation factor	Poor sleep quality
Psychological factor	
Spiritual coping on disease	Spiritual adaptation
Changes in spiritual activity	

### 4.2 Theme 1: Nursing Professionalism

Covid‐19 patients observe how nurses treat them, including whether they are viewed as a source of comfort. In this theme, two categories were identified—the fulfilment of basic needs and the attitudes of nurses.

### 4.3 The Fulfilment of the Basic Need

Patients with Covid‐19 viewed that the fulfilment of their basic needs affected their comfort when being treated in an isolation room. These basic needs included dressing, as expressed in the following quote:

‘I think to define either uncomfortable or not, hump…It might be when I requested a change of clothes. Because I understand very well that the nurse is busy. So, I ask for their help at least before sunset, right, to ask for a change of clothes or something like a body wash’. (PP 15)

The participant nurse also said that the patient became comfortable when their daily activities were facilitated by nurse. One nurse also said that basic needs such as taking a bath provide comfort for the patient.

‘The comfort is also some treatments such as bathing. For some patients need to get assistance for bathing, the others don't’. (PN 3)

### 4.4 The Attitude of Nurses

Interviews revealed that the attitudes of nurses could be a source of comfort for patients during the isolation precautions. A friendly attitude and compassion were the keywords that appeared in one of the patient quotes, wherein it was clear that this attitude was incredibly important to the patient:

‘I was comfortable since the nurse was friendly…I think that's the number one for patients outside of medicine. Indeed, it's important because [to embrace] patients with compassion’. (PP 5)

How quickly a nurse responded to a patient's call was also a determinant of comfort. A nurse's responsiveness was shown to influence patient comfort as expressed in the following quote:

‘Because I was also pregnant, my chest and stomach were very painful when I got vomited. It's very unpleasant. So, I need to be responded to quickly. That's why I feel comfortable because they came quickly every time, I needed some help. Once when I need help because I had no any vomit bag at midnight; the nurse responded so quickly. Thank God for me’. (PP 15)

### 4.5 Theme 2: Physical Discomfort

The results of the data analysis showed the patient experience of physical discomfort to be an emerging theme. Under the theme of physical discomfort, two categories were identified—namely, the clinical manifestations of disease and the impacts of treatment programmes.

### 4.6 Clinical Manifestation of Disease

The process of analysing patient interview data revealed clinical manifestations of disease; thus, these findings comprise the theme of clinical manifestations of disease as outlined below. In this category, patients expressed discomfort during treatment due to various physical manifestations of the Covid‐19 disease. These clinical manifestations included fever, shortness of breath or chills, as expressed in the following quotation:

‘That's what I feel, only hot breath, fever, cold, shivering, that's the most uncomfortable thing’. (PP 4)

In the nurse participants, also reinforce what is expressed by the patient in this category. This can be seen in the following quote:

‘their discomfort due to symptoms such as cough, fever, sore throat….’. (PN 4)

### 4.7 The Impact of the Therapy Programme

Physical discomfort is also formed from the category of the impact of the treatment programme. Patient participants revealed that the existence of various therapies gave a sense of physical discomfort. Infusion installation is one of the keywords that appear as a form of patient discomfort while being treated in the isolation room. As in the quote below:

‘Em…because I got an infusion therapy in my hand, it's difficult for the body to move. It's not comfortable. I can't move my body as usual, it's so bad. I also have small veins, which are very difficult to find’. (PP 16)

The nurse participants also underscored the discomfort of treatment therapies, especially the side effects of the oral drugs consumed by patients during treatment:

‘Therapy from the doctor made discomfort—things such as the smell of the N‐Acethylcystein capsule. Its smell is so uncomfortable for patients, so they complain that they got nausea. There was also discomfort experienced due to IV catheter insertion’. (PN 5)

### 4.8 Theme 3: Psychological Responds

Psychological responses appeared as a theme in the analysis of the interview data. Patient participants and nurses agreed that there were psychological responses that emerged as providing a form of comfort to patients being treated in an isolation room. This theme is comprised of two categories—namely, anxiety and self‐acceptance of disease.

### 4.9 Anxiety

Patients who were treated revealed that he experienced anxiety as an inconvenience. This anxiety can come from the situation related to the patient's disease conditions. This can be seen in the following quote:

‘My anxiety was like, ‘This is going to get worse, won't it?’ I also have a lot of things on my mind. So, I felt tense and anxious’. (PP 16)

The presence of anxiety in patients was also reinforced by the statements from the nurse participants. This anxiety was found to stem from the condition of the patient's disease and what that patient is experiencing at a given time. This was shown in expressions such as the following:

‘Most patients with high‐concentration oxygen therapy also cause discomfort for them, which makes them anxious. The condition of the isolation room made everything worse’. (PN 1)

### 4.10 Self‐Acceptance of Disease

Another category of psychological responses is the self‐acceptance of illness. Patient participants were shown to have a psychological response in terms of how to accept themselves as having been exposed to Covid‐19 disease. The following quote offers an example of this:

‘When I first got Covid, to be honest, it was hard for me., I'm thinking about it in the future, I'm afraid that my lungs will have heavy spots, it will hinder my performance, so what's the name…on a small scale, it's a bit difficult to get to a hospital’. (PP 11)

‘At first, I was shocked because I didn't expect it. I don't think so because I'm also one of those who don't deserve it. But my body was not in the best condition due to exhaustion. But still, I didn't expect it‐ I didn't expect it and I was just shocked’. (PP 5)

### 4.11 Theme 4: Poor Sleep Quality

Another theme identified was a decrease in sleep quality due to discomfort. Patient participants felt discomfort impacted their sleep needs. This theme includes the following two categories: clinical manifestation factors and psychological factors.

### 4.12 Clinical Manifestation Factor

Participants of Covid‐19 patients felt a decrease in sleep quality due to clinical manifestations of the Covid‐19 disease. This category can look like in the following quote:

‘I suddenly woke up that night because I suddenly felt nauseous, so when I woke up that night, what was that uncomfortable feeling in my stomach, I was thirsty, I wanted to drink, but I was afraid of throwing up, so it was uncomfortable’. (PP 2)

Nurse participants also reinforced this category, emphasising sleep difficulties from coughing and shortness of breath. This can be seen in the following quote:

‘At midnight patients usually had difficulty sleeping due to the cough. Cough and short breath caused sleep deprivation. I usually arrange the semi‐Fowler's position so the patient can breathe easier. But sometimes it didn't work. So, I start to use oxygen therapy with a nasal cannula, and then the patient can begin to sleep’. (PN 9)

### 4.13 Psychological Factor

Psychological factors are the second category in shaping the theme of decreased sleep quality. Patient participants indicated the role of psychological conditions in decreasing sleep quality as a form of patient discomfort while being treated in the isolation room. This can be seen in the following quote:

‘I still didn't sleep well. Can sleep well when I was at home, but here I still wake up and I don't know why’. (PP 2)

This was further strengthened by the nurse participants that psychology influenced the decline in sleep quality. This can be seen in the following quote:

‘The discomfort feeling is caused by the psychological factors experienced by the patient. This means that most patients can't sleep because they think about their family at home and also not feeling well while in the room. Also, because the conditions are not comfortable in the room alone, you feel a bit anxious or afraid in the room. This condition led them to difficulty sleeping’. (PN 5)

### 4.14 Theme 5: Spiritual Adaptation

Patient comfort also was determined by the spiritual adaptation process. This theme is comprised of two categories—namely, spiritual coping with disease and changes in spiritual activity.

### 4.15 Spiritual Coping on Disease

Patient participants utilised spiritual coping in accepting the disease as they were experiencing it. This is part of the idea of spiritual comfort in patients. The following interview excerpt shows the existence of a spiritual coping mechanism against illness:

‘we want to be healthy, it's just that humans are weak creatures, now God shows his power. When I got this disease, that means there is no power except Allah. The world doesn't care how strong we are, we can still be sick. That's the way of God shows His power’. (PP 1)

Nurse participants also strengthened the presence of spiritual coping in COVID‐19 patients. This can be seen in the following quote.

‘I could see the patient somehow was so tough even though they got infusion many times, but I don't know whether it was influenced by their worship or something else. They just said “I'm okay” with full calmness. On the other hand, I also noticed some patients who didn't do many worships. I found them seemed like not so comfortable while in an isolation room’. (PN9)

### 4.16 Changes in Spiritual Activity

Changes in spiritual activity make up another category under the theme of spiritual adaptation. Data analysis showed a change in spiritual activity that occurred during treatment in the isolation room. This can be seen in the following nurse participant quote:

‘I do many things to calm myself down when I'm worried, such as reciting the Qur'an and praying a lot. That's what I do regularly here now’. (PP 15)

This was reinforced by another nurse participant, in which a nurse observed a change in a patient's worship activities as follows:

‘I think their prayers were more punctual than usual. It's probably because they were busy with their job before they got sick, and I think it's the right time to get closer to God’. (PN 1)

## 5 Discussion

This study showed that comfort is a major concern for patients with Covid‐19 during isolation precautions undertaken in the hospital setting. It has been postulated that comfort is an essential nursing outcome as well as a basic human need (Kolcaba 1994). The atmosphere of isolation rooms in conjunction with Covid‐19 characteristics affects how patients perceive their comfort levels and the role of nursing care is essential to meet their comfort needs. This notion is supported by the five themes of the present study. These themes all describe the experiences of patient comfort and how patients obtained their desired comfort levels.

The findings under the theme of nursing professionalism proved that nursing care plays a pivotal part in achieving patient comfort. This result is consistent with Kolcaba's theory, which explicates the enhancement of comfort is a result of nursing intervention and the importance of comfort as a nursing outcome (Kolcaba 1994). The types of nursing interventions that meet a patient's basic needs are therefore a way to improve experiences of patient comfort (Ribeiro, Marques, and Ribeiro 2017). The restriction of visitors or caregivers from isolation rooms led to a higher demand for nurses to take care of meeting patients' basic needs, such as grooming and other hygiene procedures. Thus, it can be presumed that the participants of this study felt grateful when such needs were fulfilled by nurses properly.

The informants also revealed that the attitudes of nurses during interactions with patients are imperative to bringing comfort to patients. Nurses being friendly, compassionate and helpful was considered by informants as key to their experiences of comfort. This finding was also supported by a previous study that showed the experience of comfort is constructed through positive interactions between nurses and patients (Pott et al. 2013).

The association of comfort and interaction has been explicated by nursing theorists such as Hildegard Peplau and Imogene King who emphasised the importance of quality interpersonal relationships for providing comfort (Forchuk 1991; King 1981). In isolation rooms, the demand for interaction is deemed higher due to visitor restriction and psychological problems such as depression, anxiety and loneliness (Aungsuroch, Juanamasta, and Gunawan 2020; Mazza et al. 2020; Zandifar et al. 2020). These circumstances lead informants to rely on their interaction with nurses to meet their comfort needs. Therefore, the positive attitude of nurses during interaction is essential.

Physical discomfort remains a challenge in caring for hospitalised patients, which was shown in the present study. Covid‐19 clinical manifestations such as fever, cough, dyspnoea, fatigue and sore throat were reported by informants as discomforting experiences during admission, which also occurred in previous studies (Alimohamadi et al. 2020; Lovato and De Filippis 2020). Additionally, hospital care routines such as IV catheter insertion contributed to the informants' experience of discomfort. This issue has also been explicated in previous qualitative studies (Larsen et al. 2017; Plohal 2021). These clinical manifestations and IV catheter insertion have been the source of discomfort that needs to obtain the attention of nurses in providing nursing care. Patient comfort as an outcome of nursing intervention is indisputable.

Psychological responses are another way that patients may express their comfort and discomfort. Anxiety is a major concern, often perceived as a form of discomfort in patients that manifests during isolation precautions. Indeed, anxiety was the most frequent psychological problem that occurred in hospitalised patients with Covid‐19 in several previous studies (Mazza et al. 2020; Moayed et al. 2021; Zandifar et al. 2020). However, this study revealed that various factors may cause anxiety, not simply the characteristics of the disease itself, such as family matters and loneliness due to quarantine (Alimohamadi et al. 2020; Hsiao et al. 2021).

Furthermore, the clinical conditions of a patient's roommate can also have psychological impacts because shared isolation rooms allow every patient to see each other. In Indonesia, shared isolation room was a common type of isolation room during the Covid‐19 pandemic. The main benefit of this type is that it provides more beds than a single room. However, this room produced a significant stressor for patients particularly when a roommate has a severe condition or dies.

The impact of isolation rooms on emotional well‐being has been evaluated in several previous studies (Hossain, Sultana, and Purohit 2020; Purssell, Gould, and Chudleigh 2020; Sharma et al. 2020). However, those studies evaluated the type of single isolation room that cause loneliness and fear felt in patient during contact isolation (Hossain, Sultana, and Purohit 2020; Lupión‐Mendoza et al. 2015).

Sleep disturbance was another form of discomfort reported in this work. Several previous quantitative studies have also reported that hospitalised patients with Covid‐19 had sleep problems (Jiang et al. 2020). However, the present study emphasised clinical manifestations and psychological conditions as the main causes of sleep disturbance in such patients. This finding has been supported by previous studies of patients without Covid‐19, which found there was a strong correlation between physical and psychological problems and sleep deprivation (Wesselius et al. 2018).

The spiritual dimension is another domain that requires attention in patients with Covid‐19 during quarantine. This work has thus shown the experiences of comfort that may be associated with spiritual coping. Patients with Covid‐19 were found to feel more connected to God and have an increased need to pray (Rahimaghaee, Vizheh, and Hatamipour 2022). Therefore, the spiritual needs of Covid‐19 patients in isolation are a major concern in holistic nursing care −19 (Ferrell et al. 2020). Furthermore, this notion provokes the need for comfort from a spiritual perspective as a necessary part of holistic comfort (Kolcaba 1994).

This work provides further evidence of the importance of comfort in terms of basic human needs being met and the outcomes of treatment interventions. Hospitalised patients with Covid‐19 were discontent during isolation precautions and emphasised their physical and psycho‐spiritual experiences. This situation reflects the existence of comfort needs in a holistic approach. A definition of holistic comfort has been proposed by Kolcaba as the outcomes of nursing interventions that encompass physical, psycho‐spiritual, social‐cultural and environmental factors (Kolcaba 1992, 1994). Clinical manifestations and characteristics of Covid‐19 and the Covid‐19 pandemic were found to play pivotal roles in altering the comfort of hospitalised patients in the present study. During the data‐collection period, people in Indonesia were still haunted by the Delta variant that had caused thousands of deaths in June and July 2021 (Kemenkes 2021). This history remained prevalent in informants' minds in the present work. Therefore, their experiences of discomfort were not only caused by clinical signs of Covid‐19 but also the effects of the pandemic overall.

Additionally, the author offers the notion that comfort experiences are also affected by the environment of isolation rooms. The environment is a domain mentioned in holistic comfort in Kolcaba's work (Kolcaba 1992, 1994). In this context, the environmental theory is essential in providing the best quality of nursing care, as introduced by Florence Nightingale in the second half of the nineteenth century (Medeiros, Enders, and Lira 2015). In the context of isolation rooms, several previous works have reported on the side effects of such isolation, encompassing psychological problems, sleep disturbances and patient satisfaction levels (Guilley‐Lerondeau et al. 2017; Sharma et al. 2020). The nuances of a given room and the restriction of family visitors have been found to induce worsening psychological conditions. Similar circumstances are presumed to have occurred in the participants of the present study. Therefore, it can be said that the characteristics of isolation rooms affect experiences of comfort.

This work has revealed that comfort should not be considered merely in the physical context but it should also be viewed as including psychological and spiritual needs to be addressed by healthcare providers. All instances of discomfort were also found to be in alignment with Kolcaba's theory of holistic comfort. However, this work also adds another crucial point to the discussion—the idea that nursing care is critical for patient comfort. The concept of comfort has been discussed over the last three decades as a nursing outcome, nursing process and basic need (Kolcaba 1994). The role of the nurse is important in providing comfort for patients. This study showed that nurses can contribute significantly to improving the comfort experienced by patients. Thus, comfort as a nursing outcome and a basic human need can be understood as essential aspects in the provision of nursing care for hospitalised patients with Covid‐19 during isolation precautions.

### 5.1 Strengths and Limitations

The main strength of this work is the interview process included both patients and nurses as informants. However, it must be acknowledged that this study has several limitations. First, all patient participants interviewed were from the same hospital. The author also has concerns that the facilities of the hospital may have affected the experience of patient comfort. Second, most informants were aged in their early 30s. Patients of an older age should also have been recruited to provide more variance in perceptions. Finally, although this study involved nurse participants, the characteristics of the nurses, such as age and gender, were not diverse. These limitations should be considered in generalising the results. However, this study can still be beneficial in providing additional data in making regulations regarding the competence of nurses in isolation care.

## 6 Conclusion

This study confirmed that patients with Covid‐19 experienced discomfort during isolation precautions. The comfort disturbance involved physical, psychological, social and spiritual aspects. However, this study also revealed that a pivotal source of comfort is provided via nursing care. The informants reported that interventions by nurses increased their comfort levels during isolation precautions. Hence, nursing interventions are essential to improving patient satisfaction during isolation care.

The findings highlight the need for improvement in nursing competence to assess the comfort need and improve the comfort level of patient in isolation room. Therefore, the preparation of nurses to understand comfort need and the impact of isolation room should be required. It is recommended that the nursing department of hospital conduct short training to enhance the nursing competence of nursing staff especially in meeting comfort need of patients in isolation room.

## Author Contributions

C.E.: conceptualization, methodology, software, formal analysis, investigation and writing – original draft. A.W.: methodology, formal analysis, writing – review and editing, validation and supervision. S.Y.: methodology, formal analysis and validation. T.E.: validation and formal analysis.

## Conflicts of Interest

The authors declare no conflicts of interests.

## Data Availability

The authors have nothing to report.

## References

[nop270075-bib-0001] Alimohamadi, Y. , M. Sepandi , M. Taghdir , and H. Hosamirudsari . 2020. “Determine the Most Common Clinical Symptoms in COVID‐19 Patients: A Systematic Review and Meta‐Analysis.” Journal of Preventive Medicine and Hygiene 61, no. 3: E304–E312. 10.15167/2421-4248/jpmh2020.61.3.1530. 33150219 PMC7595075

[nop270075-bib-0002] Aungsuroch, Y. , I. G. Juanamasta , and J. Gunawan . 2020. “Experiences of Patients With Coronavirus in the COVID‐19 Pandemic Era in Indonesia.” Asian Journal for Public Opinion Research 8, no. 3: 377–392. 10.15206/ajpor.2020.8.3.377.

[nop270075-bib-0003] CDC . 2020. “Coronavirus Disease 2019 (Covid‐19).” Retrieved 25 October. https://www.cdc.gov/coronavirus/2019‐ncov/cdcresponse/about‐COVID‐19.html.

[nop270075-bib-0004] Ferrell, B. R. , G. Handzo , T. Picchi , C. Puchalski , and W. E. Rosa . 2020. “The Urgency of Spiritual Care: COVID‐19 and the Critical Need for Whole‐Person Palliation.” Journal of Pain and Symptom Management 60, no. 3: e7–e11. 10.1016/j.jpainsymman.2020.06.034. PMC7332903 32629084

[nop270075-bib-0005] Forchuk, C. 1991. “Peplau's Theory: Concepts and Their Relations.” Nursing Science Quarterly 4, no. 2: 54–60. 10.1177/089431849100400205. 2034417

[nop270075-bib-0006] Graneheim, U. H. , and B. Lundman . 2004. “Qualitative Content Analysis in Nursing Research: Concepts, Procedures and Measures to Achieve Trustworthiness.” Nurse Education Today 24, no. 2: 105–112. 10.1016/j.nedt.2003.10.001. 14769454

[nop270075-bib-0007] Guilley‐Lerondeau, B. , C. Bourigault , A. C. Guille des Buttes , G. Birgand , and D. Lepelletier . 2017. “Adverse Effects of Isolation: A Prospective Matched Cohort Study Including 90 Direct Interviews of Hospitalized Patients in a French University Hospital.” European Journal of Clinical Microbiology & Infectious Diseases 36, no. 1: 75–80. 10.1007/s10096-016-2772-z. 27612471

[nop270075-bib-0008] Hossain, M. M. , A. Sultana , and N. Purohit . 2020. “Mental Health Outcomes of Quarantine and Isolation for Infection Prevention: A Systematic Umbrella Review of the Global Evidence.” Epidemiology and Health 42: e2020038. 10.4178/epih.e2020038. 32512661 PMC7644933

[nop270075-bib-0009] Hsiao, C. T. , J. J. Sun , Y. H. Chiang , H. L. Chen , and T. Y. Liu . 2021. “Experience of Patients With COVID‐19 in Hospital Isolation in Taiwan.” Nursing & Health Sciences 23, no. 4: 888–897. 10.1111/nhs.12878. 34468066 PMC8662058

[nop270075-bib-0010] Jadmiko, A. W. , T. N. Kristina , U. Sujianto , Y. W. Prajoko , L. Dwiantoro , and A. P. Widodo . 2022. “A Quasi‐Experimental of a Virtual Reality Content Intervention for Level of Comfort of an Cancer Patients.” Computers, Informatics, Nursing 40, no. 12: 841–847. 10.1097/CIN.0000000000000953. 35970769

[nop270075-bib-0011] Jiang, Z. , P. Zhu , L. Wang , et al. 2020. “Psychological Distress and Sleep Quality of COVID‐19 Patients in Wuhan, a Lockdown City as the Epicenter of COVID‐19.” Journal of Psychiatric Research 136: 595–602. 10.1016/j.jpsychires.2020.10.034. 33153759 PMC7590814

[nop270075-bib-0012] Kemenkes . 2021. “Perkembangan Kasus Covid‐19.” Retrieved 28 October. https://covid19.go.id/id/peta‐sebaran.

[nop270075-bib-0013] King, I. M. 1981. A Theory for Nursing: Systems, Concepts, Process. Delmar, Michigan: New York, John Wiley & Sons.

[nop270075-bib-0014] Kolcaba, K. Y. 1992. “Holistic Comfort: Operationalizing the Construct as a Nurse‐Sensitive Outcome.” Advances in Nursing Science 15, no. 1: 1–10. 10.1097/00012272-199209000-00003 1519906

[nop270075-bib-0015] Kolcaba, K. Y. 1994. “A Theory of Holistic Comfort for Nursing.” Journal of Advanced Nursing 19, no. 6: 1178–1184. 10.1111/j.1365-2648.1994.tb01202.x. 7930099

[nop270075-bib-0016] Larsen, E. , S. Keogh , N. Marsh , and C. Rickard . 2017. “Experiences of Peripheral IV Insertion in Hospital: A Qualitative Study.” British Journal of Nursing 26, no. 19: S18–S25. 10.12968/bjon.2017.26.19.S18. 29068742

[nop270075-bib-0017] Lincoln, Y. S. , and E. G. Guba . 1985. Naturalistic Inquiry. London, UK: Sage.

[nop270075-bib-0018] Loiselle, C. G. , and J. P. McGrath . 2011. Canadian Essential of Nursing Research. New York, NY: Lippincot Williams and Walkin.

[nop270075-bib-0019] Lovato, A. , and C. De Filippis . 2020. “Clinical Presentation of COVID‐19: A Systematic Review Focusing on Upper Airway Symptoms.” Ear, Nose & Throat Journal 99, no. 9: 569–576. 10.1177/0145561320920762. 32283980

[nop270075-bib-0020] Lupión‐Mendoza, C. , M. J. Antúnez‐Domínguez , C. González‐Fernández , C. Romero‐Brioso , and J. Rodriguez‐Bano . 2015. “Effects of Isolation on Patients and Staff.” American Journal of Infection Control 43, no. 4: 397–399. 10.1016/j.ajic.2015.01.009. 25721058

[nop270075-bib-0021] Mazza, M. G. , R. De Lorenzo , C. Conte , et al. 2020. “Anxiety and Depression in COVID‐19 Survivors: Role of Inflammatory and Clinical Predictors.” Brain, Behavior, and Immunity 89: 594–600. 10.1016/j.bbi.2020.07.037. 32738287 PMC7390748

[nop270075-bib-0022] Medeiros, A. B. A. , B. C. Enders , and A. L. B. C. Lira . 2015. “The Florence Nightingale's Environmental Theory: A Critical Analysis.” Escola Anna Nery 19: 518–524. 10.5935/1414-8145.20150069.

[nop270075-bib-0023] Moayed, M. S. , A. Vahedian‐Azimi , G. Mirmomeni , et al. 2021. “Depression, Anxiety, and Stress Among Patients With COVID‐19: A Cross‐Sectional Study.” In Clinical, Biological and Molecular Aspects of COVID‐19, 229. Cham, Switzerland: Springer. 10.1007/978-3-030-59261-5_19. 33656727

[nop270075-bib-0024] Noor, S. , I. Maria , and A. Agianto . 2016. “The Relationship Between Caring, Comfort, and Patient Satisfaction in the Emergency Room, Ratu Zalecha Hospital, South Kalimantan, Indonesia.” Belitung Nursing Journal 2, no. 6: 156–163.

[nop270075-bib-0025] Nural, N. , and S. Alkan . 2018. “Identifying the Factors Affecting Comfort and the Comfort Levels of Patients Hospitalized in the Coronary Care Unit.” Holistic Nursing Practice 32, no. 1: 35–42. 29210876 10.1097/HNP.0000000000000245

[nop270075-bib-0026] Plohal, A. 2021. “A Qualitative Study of Adult Hospitalized Patients With Difficult Venous Access Experiencing Short Peripheral Catheter Insertion in a Hospital Setting.” Journal of Infusion Nursing 44, no. 1: 26–33. 10.1097/NAN.0000000000000408. 33394871

[nop270075-bib-0027] Pott, F. S. , T. Stahlhoefer , J. V. C. Felix , and M. J. Meier . 2013. “Comfort and Communication Measures in Nursing Caring Actions for Critically Ill Patients.” Revista Brasileira de Enfermagem 66: 174–179. 10.1590/S0034-71672013000200004. 23743835

[nop270075-bib-0028] Purssell, E. , D. Gould , and J. Chudleigh . 2020. “Impact of Isolation on Hospitalised Patients Who Are Infectious: Systematic Review With Meta‐Analysis.” BMJ Open 10, no. 2: e030371. 10.1136/bmjopen-2019-030371. PMC7044903 32075820

[nop270075-bib-0029] Rahimaghaee, F. , M. Vizheh , and K. Hatamipour . 2022. “Development and Psychometric Properties of a Spiritual Needs Assessment Scale for Patients With COVID‐19.” Journal of Psychosocial Nursing and Mental Health Services 60, no. 4: 47–54. 10.3928/02793695-20211014-01. 34677116

[nop270075-bib-0030] Ribeiro, P. C. P. S. V. , R. M. D. Marques , and M. P. Ribeiro . 2017. “Geriatric Care: Ways and Means of Providing Comfort.” Revista Brasileira de Enfermagem 70: 830–837. 10.1590/0034-7167-2016-0636. 28793115

[nop270075-bib-0031] Sharma, A. , D. R. Pillai , M. Lu , et al. 2020. “Impact of Isolation Precautions on Quality of Life: A Meta‐Analysis.” Journal of Hospital Infection 105, no. 1: 35–42. 10.1016/j.jhin.2020.02.004. 32059996

[nop270075-bib-0032] Vaismoradi, M. , H. Turunen , and T. Bondas . 2013. “Content Analysis and Thematic Analysis: Implications for Conducting a Qualitative Descriptive Study.” Nursing & Health Sciences 15, no. 3: 398–405. 23480423 10.1111/nhs.12048

[nop270075-bib-0033] Wesselius, H. M. , E. S. Van Den Ende , J. Alsma , et al. 2018. “Quality and Quantity of Sleep and Factors Associated With Sleep Disturbance in Hospitalized Patients.” JAMA Internal Medicine 178, no. 9: 1201–1208. 10.1001/jamainternmed.2018.2669. 30014139 PMC6142965

[nop270075-bib-0034] Yücel, Ş. Ç. , G. G. Arslan , and H. Bagci . 2020. “Effects of Hand Massage and Therapeutic Touch on Comfort and Anxiety Living in a Nursing Home in Turkey: A Randomized Controlled Trial.” Journal of Religion and Health 59, no. 1: 351–364. 10.1007/s10943-019-00813-x. 30982141

[nop270075-bib-0035] Zandifar, A. , R. Badrfam , S. Yazdani , et al. 2020. “Prevalence and Severity of Depression, Anxiety, Stress and Perceived Stress in Hospitalized Patients With COVID‐19.” Journal of Diabetes & Metabolic Disorders 19: 1–8. 10.1007/s40200-020-00667-1. 33145259 PMC7594988

